# Three-Dimensional Echocardiography in Evaluating LA Volumes and Functions in Diabetic Normotensive Patients without Symptomatic Cardiovascular Disease

**DOI:** 10.1155/2020/5923702

**Published:** 2020-08-19

**Authors:** Mohamed Hamza, Ahmed Mamdouh, Dina Ezzeldin, Adnan Tawfik, Ahmed Nayel

**Affiliations:** ^1^Department of Cardiology, Ain Shams University, Cairo, Egypt; ^2^Kalar General Hospital, Sulaimania, Iraq

## Abstract

**Background:**

Cardiovascular complications are the most serious threat to diabetic patients. Associated metabolic and microvascular changes are the main cause of cardiac function affection, and the earliest cardiac change is diastolic dysfunction. Assessment of LA function changes is a key to determine early heart damage of diabetic patients.

**Objectives:**

To evaluate the effect of diabetes mellitus on left atrial volumes and functions by using real-time 3-dimensional echocardiography in normotensive patients free from cardiovascular disease.

**Methods:**

The study included 110 individuals, 50 controls and 60 patients with diabetes mellitus, 30 patients with type 1 diabetes mellitus and 30 patients with type 2 diabetes mellitus. 2-dimensional echocardiography was used to assess the LA maximum volume and LA phasic volumes, and LA maximum volume indexed to body surface area were measured by 3D echocardiography. LA functions (LA total stroke volume, LA active stroke volume, and LA active emptying fraction) were obtained from RT3D volumetric analysis.

**Results:**

The results of the analysis revealed that type 2 diabetes mellitus showed enlarged *V*_max_, *V*_min_, and LAVi with an increased LA total stroke volume and decreased active emptying fraction, while type 1 diabetics showed only decreased in active emptying fraction. The LA maximum volume indexed to body surface area (LAVi) was significantly higher in type 2 diabetic patients as compared to normal controls which was 23.55 ± 3.37 ml/m^2^ versus 20.30.

**Conclusion:**

Patients with type 2 diabetes mellitus have an increased LA volume with impaired compliance and contractility, while patients with type 1 diabetes mellitus have only impaired contractility compared to nondiabetic subjects.

## 1. Background

The quantification of the cardiac chamber size and function is the cornerstone of cardiac imaging, with echocardiography being the most commonly used noninvasive modality because of its unique ability to provide real-time images of the beating heart with high temporal and spatial resolution, combined with its availability and portability [1]. [2]

Using the advanced echocardiographic techniques, such as strain (S), Doppler, speckle tracking, and 3D echocardiography, we are able to recognize early atrial dysfunction, before clinical manifestations and earlier than standard echocardiographic parameters. [3]

Cardiovascular complications are the most serious threat of diabetes to diabetic patients. The associated metabolic and microvascular changes are the main cause of cardiac function affection, and the earliest cardiac change of diabetes is diastolic dysfunction. [4] Assessment of LA function changes is a key to determine early heart damage of diabetic patients [5].

Two-dimensional echocardiography has been the most commonly used diagnostic modality for assessing the LA size and function in daily clinical situations. However, the measurement of the LA volume is difficult due to its complex shape.

Several methods which use various geometric assumptions about the atrial shape have been developed for assessing the LA volume, such as the biplane area length (AL), the biplane modified Simpson, and the prolate ellipse methods [6].

A volumetric system developed at Duke University enabled the first real-time acquisitions. Consequently, RT3DE was used in recent studies to evaluate the LA size and/or function in various diseases [7].

## 2. Aim

The study is aimed at evaluating the effect of diabetes mellitus on left atrial volumes and functions by using real-time three-dimensional echocardiography in normotensive patients free of symptomatic cardiovascular disease.

## 3. Methods

The study was a case control study and included 110 individuals, 50 normal healthy subjects regarded as control and 60 patients with diabetes mellitus, 30 patients with type 1 diabetes mellitus and 30 patients with type 2 diabetes mellitus.

The inclusion criteria were age above 18 years, normal blood pressure, left ventricular ejection fraction ≥ 50% (modified Simpson method), sinus rhythm, and with no history of previous cardiac symptoms.

The diabetic patients included were already diagnosed as either type 1 or type 2 DM, were taking their antidiabetic medication, and were regularly visiting the diabetes clinic for follow-up.

Patients with hypertension (previously diagnosed, on antihypertensive medications, or measured BP > 140/90), structural heart disease (valvular and congenital heart disease, LVH, and impaired LV systolic functions), ischemic heart disease (by history of revascularization or documented evidence of ischemia), and arrhythmias and patients with chronic kidney disease and chronic obstructive lung diseases were all excluded from the study.

The control group consisted of 50 healthy individuals matched for age and sex with the diabetic patients.

After a written informed consent, all study participants had meticulous clinical assessment (history and physical examination) with calculation of the estimated BSA (cm^2^), ECG, and echo study.

All echocardiographic examinations were performed by the same echocardiographer, by using IE33, Philips machine, with digital storage software for offline analysis [8].

LV internal dimensions, wall thickness, and LA diameter were obtained from the parasternal long-axis view [9], and LV ejection fraction was calculated from apical four-chamber and two-chamber views using Simpson's biplane method [10] Conventional pulsed Doppler imaging of mitral inflow was recorded from apical 4-chamber view to measure the *E* and *A* waves and calculate the *E*/*A* ratio [11]. LA volumes were measured using Simpson's biplane method of discs with the LA appendage and pulmonary veins excluded from the tracing using four-chamber and two-chamber views [12].

Subsequently, all subjects performed three-dimensional echocardiographic evaluations.

Individuals were instructed to hold their breath at the time of image acquisition, and images were coupled with ECG recording. ^(197)^

The real-time three-dimensional echocardiography (RT3DE) was used to obtain the left atrial volume.

An IE33 echocardiography device, with an X3 (1-3 MHz) matrix transducer by the same echocardiographer was used to acquire the full volume, real-time pyramidal volumetric data for four consecutive cardiac cycles. To ensure the inclusion of the entire volume of the LA and LV inside the pyramidal volume, data were acquired using the wide-angle mode, thus acquiring four wedge-shaped subvolumes for a period of 5 seconds of apnea [13].

**Figure 1:**
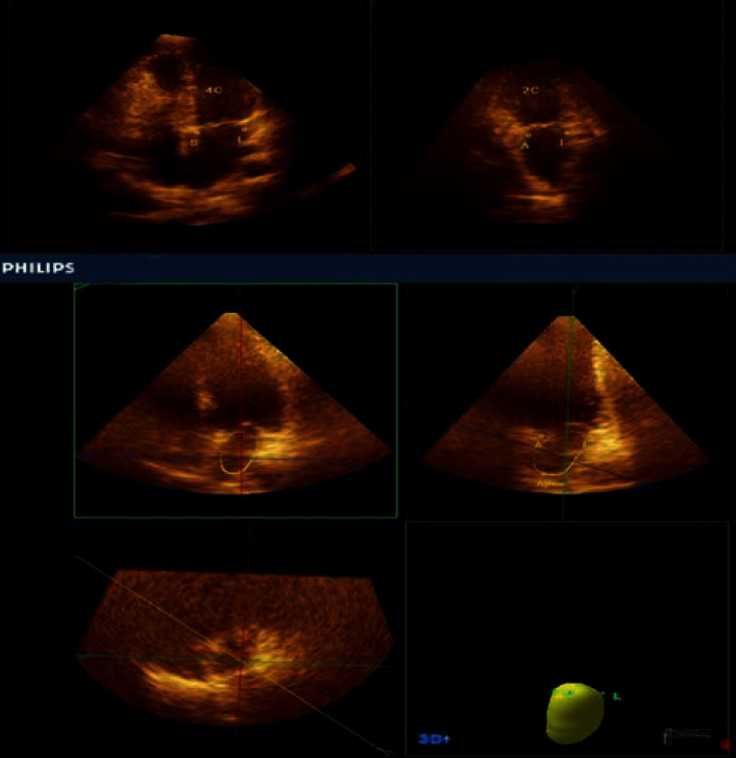
Semiautomatic LA border tracing by marking (●) at 4 mitral annular points (lateral, septal, inferior, and anterior) and an atrial superior dome point opposite the annulus A. The automatic border tracing is then shown by the software.

The echocardiographic 3D data were digitally stored and analyzed using the QLAB-Philips software (version 6.0; Philips Medical Systems). The three-dimensional echocardiographic image analysis was based on the apical window and semiautomatic tracing of the endocardial borders (Figure 1). [14]

The analysis of the left atrium was performed using MPR mode by marking four points on the atrial surface of the mitral annulus, septal, lateral, anterior, and inferior, and a fifth point in the left atrial roof. Subsequently, the endocardial surface was automatically outlined and could be visualized from different views [15].

Manual modifications were made to correct the automatic tracing when necessary, and then, the software generates a variation curve of the LAV throughout the cardiac cycle [16]. The LA appendage and the pulmonary vein confluence were excluded from the LA tracings, and finally, the dynamic LA polyhedron model was obtained [17].

The LAV_max_ was considered the peak of the curve, and the value was indexed by the body surface area (LAVi) [18]..

**Figure 2:**
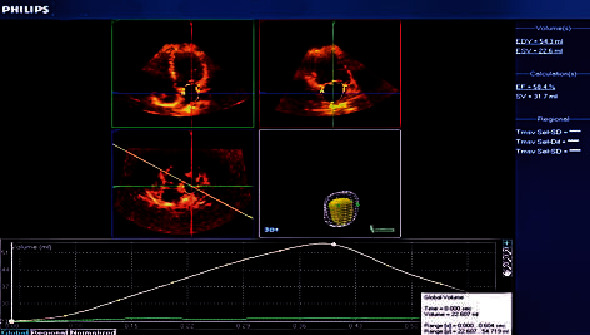
Left atrial volume by real-time three-dimensional echocardiography at the end diastole (LAV_min_).

**Figure 3:**
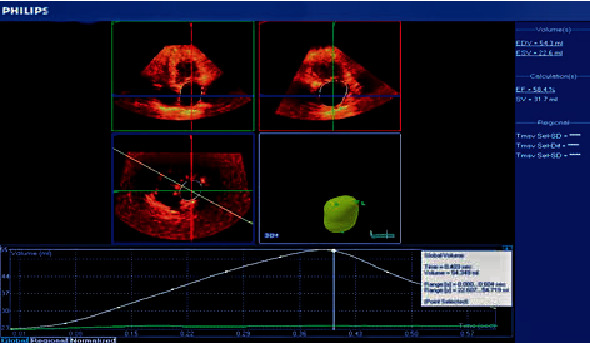
Left atrial volume by real-time three-dimensional echocardiography at the end systole (LAV_max_).

**Figure 4:**
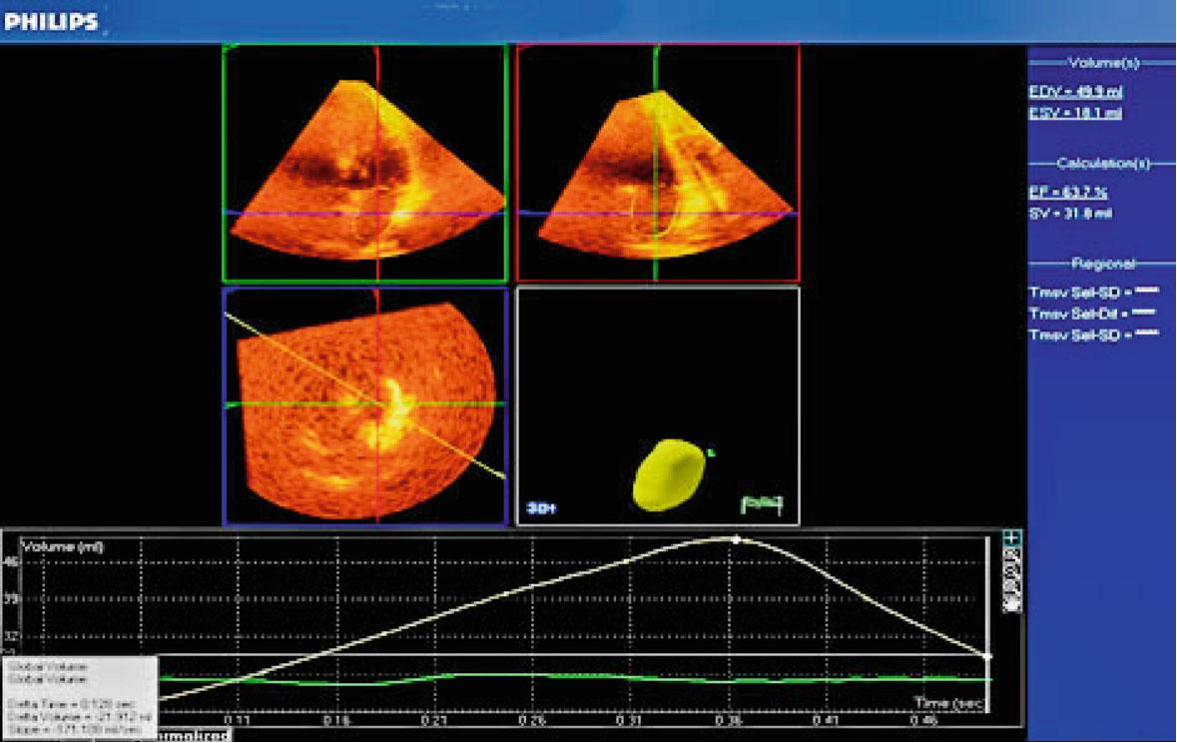
Left atrial volume by real-time three-dimensional echocardiography at last frame before mitral reopening (*V*_preA_).

The following left atrial volumes were measured: LA minimum volume (LAV_min_) and LA end-diastolic volume measured at the first frame after mitral valve closure (Figure 2) [19], LA maximum volume (LAV_max_) and LA end-systolic volume measured one frame before mitral valve opening (Figure 3) [19], and LA volume before contraction (*V*_preA_): the last frame before mitral valve reopening or at the time of P wave on ECG (Figure 4) [19].

From these volumes, the following indices of LA function were calculated: LA total stroke volume (LASV): LAV_max_–LAV_min_, LA active stroke volume (ASV): *V*_preA_ − *V*_min_, and LA active emptying fraction (AEF): ASV/*V*_preA_ × 100 [20].

## 4. Statistical Analysis

Data were collected, revised, coded, and entered to the Statistical Package for Social Science (IBM SPSS) version 20. Qualitative data were presented as number and percentages while quantitative data were presented as mean, standard deviations, and ranges.

The comparison between two groups with qualitative data were done by using the chi-squared test, and/or the Fisher exact test was used instead of the chi-squared test when the expected count in any cell was found less than 5.

The comparison between two independent groups regarding quantitative data with parametric distribution was done by using the independent *t*-test, while comparison between more than two groups with quantitative data was done by using one-way analysis of variance (ANOVA).

Spearman correlation coefficients were used to assess the relation between two quantitative parameters in the same group.

The confidence interval was set to 95%, and the margin of error accepted was set to 5%. So, a *P* value less than 0.05 was considered significant.

## 5. Results

This study included 110 individuals, 50 normal healthy subjects and 60 patients with diabetes mellitus, 30 patients with type 1 diabetes mellitus and 30 patients with type 2 diabetes mellitus. Patients were consecutively recruited from the diabetic clinic of Ain Shams University hospital. Baseline demographic data including age and sex are listed in Table 1.

The duration of diabetes in type 1 diabetic patient group ranged from 4 to 26 years with a mean of 12.60 ± 6.32, and the duration of diabetes in type 2 diabetic patients ranged from 1 to 20 years with a mean of 10.20 ± 6.19. There was no significant difference in the duration of diabetes in both subgroups (*P* > 0.05).

Regarding LV dimensions, wall thickness, and LV systolic functions, there was no statistically significant difference between the 3 groups included in the study (control subjects and type 1 and type 2 diabetic patients) (Table 2).

### 5.1. Left Atrial Dimensions and Area by 2D Echocardiography

The LA area was larger in the diabetic group (*P* < 0.05) while the LA length and diameter showed no statically significant difference between control subjects and diabetic patients (Table 3).

### 5.2. Left Atrial Volume by Two-Dimensional Echocardiography

The patient group shows a significantly larger left atrial volume in comparison to control group (*P* < 0.005) (Table 4).

After dividing the patient group into diabetic subgroups (type 1 and 2), there was no statistically significant difference between controls and diabetic type 1 regarding LA volumes but type 2 diabetic patients showed a statistically significant higher LA volumes when compared to controls.

There was a statistically significant difference between both study groups regarding mitral *E*/*A* ratio and *A* velocity (*P* < 0.05).

Regarding the mitral inflow velocity (*E*/*A*) ratio, there was no significant difference between control subjects and type 1 diabetics, but on the other hand, there was a statistically significant difference between controls and type 2 diabetes patients.

We studied 30 cases with diabetes mellitus type 1 in which 16.7% had left ventricular diastolic dysfunction grade 1 (*E*/*A* < 1) and 30 cases with diabetes mellitus type 2 in which 18 cases (60%) had left ventricular diastolic dysfunction grade 1 (*E*/*A* < 1).

### 5.3. Left Atrial Volume by 3D Echocardiography

The LA minimum volume, maximum volume, volume before atrial contraction, and LA volume indexed to the BSA were significantly higher in patients with type 2 diabetes *P* value < 0.05 while there was no significant difference between control subjects and patients with type 1 diabetes regarding *V*_min_, *V*_max_, *V*_preA_, and LAVi (*P* > 0.05).(Table 5).

### 5.4. Left Atrial Function by 3D Echocardiography

Patients with type 1 diabetes showed a significantly lower active emptying fraction as compared to the control group (*P* < 0.05) with no differences regarding the LA total stroke volume and LA active stroke volume between both study groups (*P* > 0.05) while patients with type 2 diabetes showed a significantly larger LA total stroke volume and lower LA active emptying fraction (*P* < 0.05) with no difference regarding the LA active stroke volume (*P* > 0.05) when compared to control subjects(Table 6).

Patients with type 2 diabetes showed a significantly larger LA total stroke volume as compared to those with type 1 diabetes (*P* < 0.05) with no differences in LA active stroke volume and LA active emptying fraction (*P* > 0.05) (Table 7).

### 5.5. Correlation of Patient Characteristics with Left Atrial Volume at Different Phases

Age showed a strong correlation with the left atrial volume at different phases (*V*_min_ and *V*_max_) and the left atrial volume indexed to body surface area (LAVi) (*P* < 0.001, 0.001, and 0.007, respectively) but showed no correlation with *V*_preA_ volume (*P* value = 0.178).

The weight and body mass index (BMI) showed a strong correlation with *V*_max_ only (*P* value = 0.030 and 0.002, respectively.

The duration of diabetes mellitus showed no correlation with the left atrial volume at any phase (*V*_min_, *V*_max_, and *V*_preA__)_ and left atrial volume index (LAVi).

### 5.6. Correlation of 2D LA Volume, Area, and Dimensions with 3D LA Volume

The maximum left atrial volume obtained by 2D echocardiography correlated positively with the phasic left atrial volume obtained by 3D echocardiography (*V*_min_, *V*_max_, and *V*_preA__)_.

The maximum left atrial volume indexed to the body surface area (LAVi) showed positive correlation with *V*_min_ and *V*_max_ with no correlation with *V*_preA_ (Table 7).

The left atrial area (LA area) obtained by 2D echocardiography from apical four-chamber view showed a significantly positive correlation with *V*_min_, *V*_max_, and LAVi (*P* value < 0.05).

The left atrial length (LA leng.) obtained by 2D echocardiography from apical four-chamber view showed a significantly positive correlation with *V*_max_ and LAVi (*P* value < 0.05).

The left atrial diameter (maximum diameter) obtained by 2D echocardiography with M-mode from parasternal long-axis view showed a strong correlation with *V*_min_ and *V*_max_ (*P* value < 0.05) with no correlation with LAVi and *V*_preA_ (*P* value > 0.05).

The mitral inflow velocity (*E*/*A*) ratio showed a significant correlation with *V*_min_ and *V*_max_ (*P* value < 0.05) (Table 8).

The regression analysis model demonstrated that the presence of diabetes mellitus, type of diabetes mellitus, and age were independent predictors of *V*_min_ while only the type of diabetes mellitus was an independent predictor of *V*_max_; it also demonstrated that the presence of diabetes and type of diabetes were independent predictors of LAVi. Only the type of diabetes was an independent factor of the LA total stroke volume.

## 6. Discussion

The first published report of 3D echocardiographic evaluation of the LA size was that of King et al. in 1992. The authors studied 30 patients who were referred to the echocardiography laboratories for clinically indicated examinations. The patients were chosen randomly and were unselected for the type of heart disease. Each patient was required to have a technically satisfactory study to be included. The study concluded the potential usefulness of 3D echocardiography in completing 2D echocardiographic data in a comprehensive assessment of the LA size [21].

As we evaluated LV dimensions, LV systolic function, LA length, and LA anteroposterior diameter using 2D echocardiography, we did not observe any significant difference when we compared type 2 diabetic patients with normal controls; our results were in agreement with Atas et al. in 2014, who showed no significant difference regarding LV dimensions, LV systolic function, LA length, and LA anteroposterior diameter by using 2D echocardiography, when they compared the type 2 diabetic group with the normal control group [22].

As we used 2D guidance Doppler wave to interrogate mitral inflow velocities, we observed that atrial filling (*A*) wave velocity was 75.04 ± 17.82 cm/sec in type 2 diabetic patients which was significantly increased among type 2 diabetic patients when compared to normal controls, and we also observed that the *E*/*A* velocity ratio was 0.94 ± 0.30 in type 2 diabetic patients which was decreased significantly among type 2 diabetic patients as compared to normal controls.

Gul and colleagues in (2009) compared diastolic parameters in 81 type 1 diabetic patients and 50 healthy volunteers using both pulse wave Doppler and tissue Doppler imaging to evaluate the possible effects of type 1 diabetes on left ventricular dysfunction; they demonstrated that the (*A*) velocity was 63 ± 0.30 cm/sec and showed to be significantly increased in type 1 diabetic patients when compared with normal controls [23].

These results are similar to ours; in our study, the (*A*) velocity was 73.83 ± 11.74 cm/sec in type 1 diabetic patients and was significantly increased when compared to normal controls.

AThere are several methods to measure the left atrial volume using 2DE, including Simpson's biplane method of discs, biplane area length, and the prolate ellipse. The two biplane methods compare closely and are recommended as the standard for left atrial volumes in ASE guidelines. The biplane method has the most accurate 2D echocardiographic estimation of LA volumes compared with CT, providing the closest approximation to the true left atrial volume [24].

The LA maximum volume (*V*_max_) was evaluated by 2D echocardiography with different methods (4-chamber LA volume, 2-chamber LA volume, and biplane Simpson's method); we observed that the LA maximum volume was increased significantly in type 2 diabetic patients as compared to type 1 diabetic patients and normal controls by all methods of examinations used.

In 2014, Atas and colleagues examined 40 type2 diabetics and 40 normal healthy controls using RT3DE in addition to conventional 2D echocardiography to assess the LA volume and phasic function and demonstrated that LA volumes, LA maximum volume (*V*_max_) which was 40.9 ± 11.9 ml, LA minimum volume (*V*_min_) which was 15.6 ± 5.9 ml, and LA total stroke volume which was 25.8 ± 7.1 ml were higher significantly in diabetic patients, while LA active emptying fraction was 38.5 ± 13 and was significantly reduced in type 2 diabetic patients, with no significant difference between groups regarding LA active stroke volume and LA volume before atrial contraction (*V*_preA_) [22].

Our results are in agreement with Atas et al. observations; as LA volumes in type 2 diabetic patients were higher significantly in type 2 diabetic patients when compared to normal controls; *V*_max_ was 44.09 ± 9.44 ml, *V*_min_ was 17.29 ± 4.11 ml, and total stroke volume was 25.26 ± 4.14 ml; also, LA active emptying fraction was 34.40 ± 10.78 which was reduced significantly in type 2 diabetic patients. There was no difference between the groups regarding LA active stroke and *V*_preA_.

Huang and colleagues in (2010) examined left atrial function in patients with type 2 diabetes mellitus (DM). Fifty-eight type 2 DM patients as the DM group and forty healthy people as the normal control group were enrolled in their study. EUB-6500 echocardiographic imaging system with LA volume tracking was applied to display and analyze the LA volume curve imaging on LV apical two- and four-chamber views. They concluded that LA maximum volume indexed to the body surface area (LAVi) was significantly higher in the type 2 diabetic patients when compared to normal controls [5].

Our study showed that the LA maximum volume indexed to the body surface area (LAVi) was significantly larger 23.55 ± 3.37 ml/m^2^ in type 2 diabetic patients than in normal controls which was 20.30 ± 2.11 ml/m^2^, which is similar to the results of Huang et al. 2010, in spite of using RT3DE volume analysis in our study instead of the LA volume tracking method for evaluating LA volume.

Acar and colleagues in 2009 studied LA volume and function; 43 patients with type 1 diabetes mellitus and 42 controls were enrolled in their study; they concluded that LAVi, *V*_max_, *V*min, *V*_preA_, and LA total stroke volume were similar between the both groups. However, they observed a significant increase in LA active emptying fraction in their type 1 diabetic patients which was 45.7 ± 8.6 versus 36.3 ± 11.1 in normal controls [25].

In type 1 diabetic patients enrolled in our study, we observed that the LA maximum volume index, LA maximum volume, LA minimum volume, LA volume before atrial contraction, and LA total stroke volume were 21.23 ± 4.29 ml/m^2^, 35.29 ± 7.64 ml, 12.78 ± 2.12 ml, 23.02 ± 4.34 ml, and 22.91 ± 4.88 ml, respectively, in type 1 diabetic patients versus 20.30 ± 2.11 ml, 32.44 ± 6.81 ml, 12.41 ± 2.59 ml, 23.68 ± 6.16 ml, and 21.81 ± 4.04 ml, respectively, in normal controls, with no significant differences between the two groups which was in line with Acar et al. observations.

On the other hand, we found that the left atrial pump function was impaired in our type 1 diabetic patients which was opposite to Acar et al. However, our type 1 diabetic patients displayed lower active emptying fraction and similar passive emptying fraction (LA volume before atrial contraction) values compared to controls which is not an expected finding for impaired left ventricular diastolic compliance in which a compensatory increase of LA contractility and pump function is expected. Based on these findings, it may be suggested that an independent atrial cardiomyopathy associated with diabetes might also be operative on the altered left atrial volume and functions in our diabetic patients [26].

In a study by Chillo and colleagues in 2013, they performed echocardiography for 122 type 2 and 58 type 1 diabetic patients to determine the prevalence of LA enlargement and its relation to LV diastolic dysfunction among asymptomatic diabetics and they concluded that enlarged LA volume and LV diastolic dysfunction were more common in type 2 than in type 1 diabetic patients. Patients with enlarged LAVi were older when compared with patients with normal LAVi both among type 1 and type 2 diabetic groups [27].

As we compared type 1 diabetics with type 2 diabetics, we observed that LA volumes were increased in type 2 diabetic patients, and diastolic dysfunction was more common among type 2 diabetic patients; however, both type 1 and type 2 diabetics showed significantly impaired LA pumping function. This finding may indicate deterioration of active relaxation and compliance and contractility of LA myocardium in diabetic patients. Although the mechanism of impairment is not clear, injury to atrial myocardium caused by sustained hyperglycemia and fibrotic alteration of LA have been suggested to be contributing factors; age and nature of disease may be the cause for the difference between the 2 types of diabetes.

When we compared the 2D echocardiographic LA area with 2D and 3D LA volume, there was a significantly positive correlation and insignificant difference; our results were explained by an abstract titled “Comparison of left atrial size by freehand scanning three-dimensional echocardiography and two-dimensional echocardiography” reported by Kawai and colleagues in 2004. The 2D LA area and 2D LA volume showed a significant positive correlation with the 3D LA volume [28].

The anteroposterior diameter of LA measured using 2D-guided M-mode from parasternal long-axis view showed no correlation with LA volume, and this was explained previously by Lester et al. LA dilatation might not be evenly distributed in all planes, and measurement of anteroposterior dimension is likely to be insensitive to changes in LA size [29].

In our diabetic patients, 80% of type 1 and 40% of type 2 diabetic patients were classified as having normal diastolic function (*E*/*A* ≥ 1); interestingly, LA enlargement was present in this group as well. These observations are similar to those reported by Jarnert et al. In that study, LAVi was increased even in the subgroup with normal diastolic function. This suggests that an altered LV diastolic function in DM only contributes in part to observed LA changes. Thus, it is likely that an independent atrial cardiomyopathy associated with DM may be a likely contributor to LA enlargement [30].

Zhong et al. performed a study using RT3DE in healthy subjects to calculate LAVi, LA volume maximum (*V*_max_), and LA volume minimum (*V*_min_) which increased with age. Similarly, we found that LAVi, *V*_max__,_ and *V*_min_ were positively correlated with age [31].

Gardts and colleagues in 2002 assessed the left atrial volume in 941 hypertensive patients with a mean age 66 years using 2D echocardiography; they concluded that the LA volume was positively correlated with body mass index and age [32].

The present study was designed to assess the effects of diabetes mellitus (type 1 and type 2) on left atrial size and function and showed that the LA volume was increased and LA mechanical function was impaired in type 2 diabetic patients, whereas type 1 diabetic patients only showed impaired LA pump function.

## 7. Study Limitations

This was a cross-sectional study, and the prognostic importance of our findings is not clear. The sample size was small, and further studies on a larger number of patients are needed.

The LA appendage has an important role for the function and volume measurements of LA, but it was excluded in the study which may have affected the results.

Software Q Lab Philips version 6 was used for the analysis of 3D volumetric data which is a relatively old version of the available software and is originally designed for evaluation of left ventricular volumes. However, using this software for evaluation of LA volumes also seems to be prudent as it has been used by many other studies in the literature. As to our knowledge, there is no dedicated LA software available in the market.

## 8. Conclusion

1. Evaluation of asymptomatic diabetic patients by using RT3DE atrial volume analysis may facilitate recognition of subtle alterations related with type 1 and type 2 diabetes
2. Patients with type 2 diabetes mellitus have increased left atrial volume and impaired left atrial compliance and contractility in relation to normal healthy subjects
3. Patients with type 1 diabetes mellitus have impaired left atrial contractility in relation to normal healthy subjects
4. The presence of diabetes mellitus, type of diabetes mellitus, and age were independent predictors of *V*_min_ while only the type of diabetes mellitus was the independent predictor of *V*_max_
5. The presence of diabetes mellitus and type of diabetes mellitus were independent predictors of LAVi, and only the type of diabetes mellitus was the independent predictor of LA total stroke volume while none of them were independent predictors of LA active emptying fraction
6. Intrinsic alterations in atrial myocardial activity seem to be responsible for left atrial dysfunction in addition to impairment in left ventricular diastolic function which is known to be common in diabetic patients

## Figures and Tables

**Table 1:** Baseline demographic data.

		Control group	D1 group	D2 group
Age (year)	Mean ± SD	33.63 ± 8.57	25.53 ± 5.88	51.97 ± 9.33
	Range	18–58	18–38	37–70

Sex	Female	17 (34%)	24 (80%)	22 (73.3%)
	Male	33 (66%)	6 (20%)	8 (26.7%)

Weight (kg)	Mean ± SD	74.65 ± 13.58	61.87 ± 8.80	72.40 ± 8.02
	Range	50–95	45–80	55–90

BMI (kg/m^2^)	Mean ± SD	25.45 ± 3.26	23.32 ± 2.90	26.26 ± 2.79
	Range	18.4–29.7	16.5–28.1	20.2–29.4

Weight (kg)	Mean ± SD	74.65 ± 13.58	61.87 ± 8.80	72.40 ± 8.02
	Range	50–95	45–80	55–90

Duration of diabetes (year)	Mean ± SD		12.60 ± 6.32	10.20 ± 6.19
	Range		4–26	1–20

**Table 2:** Comparison between patient and control groups regarding LV dimensions and function.

		Control group	Patients group	Independent *t*-test	
		No. = 50	No. = 60	*t*	*P* value
LVEDd (mm)	Mean ± SD	41.68 ± 4.41	42.29 ± 4.44	0.755	0.451
	Range	35–51	33–63		

LVESd (mm)	Mean ± SD	29.06 ± 2.05	28.98 ± 3.41	0.165	0.868
	Range	25–34	22–38		

IVS thickness (mm)	Mean ± SD	9.09 ± 0.89	9.68 ± 0.74	3.948	0.001
	Range	5–10.3	8-10.3		

LVEF%	Mean ± SD	66.15 ± 4.64	64.83 ± 5.79	1.378	0.171
	Range	60–74	53-77		

**Table 3:** Comparison between the control group and the patient group regarding LA area and dimensions by 2D.

		Control group	Patient group	Independent *t*-test	
				*t*	*P* value
LA area (cm^2^)	Mean ± SD	13.34 ± 2.29	14.05 ± 2.94	3.455	0.001
	Range	10–19.1	11.43–21.2		

LA length (cm)	Mean ± SD	4.79 ± 0.63	5.84 ± 5.11	0.695	0.489
	Range	3.7–6.2	3–6.1		

LA diameter (mm)	Mean ± SD	32.71 ± 2.68	32.68 ± 2.85	1.281	0.203
	Range	27–39	28–38		

**Table 4:** Comparison between patient and control groups regarding 2D LA volumes.

		Control group	Patients group	Independent *t*-test	
		No. = 50	No. = 60	*t*	*P* value
4C LA volume (ml)	Mean ± SD	29.20 ± 6.51	33.74 ± 6.75	3.57	0.001
	Range	20–45	12–61		

2C LAV (ml)	Mean ± SD	31.45 ± 8.15	35.79 ± 6.98	3.009	0.003
	Range	20–50	12–61		

2C LAV (bi plane) (ml)	Mean ± SD	31.85 ± 7.15	36.17 ± 6.89	3.219	0.002
	Range	24–50	14-61		

**Table 5:** Comparison between control group and patient group regarding left atrial volumes by 3D. D1: type 1 diabetes; D2: type 2 diabetes.

		Control group	D1 group	D2 group	P1	P2	P3
*V*_min_	Mean ± SD	12.41 ± 2.59	12.78 ± 2.12	17.29 ± 4.11	0.511	0.001	0.009
	Range	9–17	9–20	12–30			

*V*_max_	Mean ± SD	32.44 ± 6.81	35.29 ± 7.64	44.09 ± 9.44	0.076	0.001	0.001
	Range	24–50	15–55	34–83			

*V*_preA_	Mean ± SD	23.68 ± 6.16	23.02 ± 4.34	24.53 ± 2.62	0.600	0.514	0.226
	Range	14–30	16–36	18–30			

LAVi	Mean ± SD	20.30 ± 2.11	21.23 ± 4.29	23.55 ± 3.37	0.166	0.001	0.022
	Range	13.1–28	12–32.6	19–34.2			

**Table 6:** Comparison between the control group and the patient subgroup regarding left atrial function by 3D.

		Control group	D1 group	D2 group	P1	P2	P3
LA total stroke volume	Mean ± SD	21.84 ± 4.04	22.91 ± 4.88	25.26 ± 4.14	0.275	0.001	0.049
	Range	14–29	17.1–44.2	18–33.5			

LA active stroke volume	Mean ± SD	10.02 ± 3.0	9.73 ± 2.15	9.71 ± 3.71	0.635	0.665	0.976
	Range	3.6–16	6.2–17	4–19			

LA active emptying fraction	Mean ± SD	42.85 ± 10.05	34.38 ± 11.35	34.40 ± 10.78	0.001	0.001	0.994
	Range	19.4–61.5	15–58.6	16.6–58.6			

**Table 7:** Correlation between LA volumes and demographic parameters.

	*V*_min_		*V*_max_		*V*_preA_		LAVi	
	*r*	*P* value	*r*	*P* value	*r*	*P* value	*r*	*P* value
Age	0.374^∗∗^	0.001	0.449^∗∗^	0.001	0.124	0.178	0.247^∗∗^	0.007
Duration of diabetes	-0.048	0.713	-0.066	0.617	0.060	0.647	-0.007	0.958
Weight	0.054	0.557	0.198^∗^	0.030	0.086	0.348	-0.099	0.281
BMI	0.082	0.372	0.281^∗∗^	0.002	0.023	0.800	0.017	0.856

**Table 8:** Correlation between 2D data and LA 3D volumes.

	*V*_min_		*V*_max_		*V*_preA_		LAVi	
	*r*	*P* value	*r*	*P* value	*r*	*P* value	*r*	*P* value
2C LAV	0.337^∗∗^	0.001	0.742^∗∗^	0.001	0.141	0.126	0.460^∗∗^	0.001
2C (BiP)	0.444^∗∗^	0.001	0.906^∗∗^	0.001	0.143	0.120	0.580^∗∗^	0.001
LA volume	0.469^∗∗^	0.001	0.764^∗∗^	0.001	0.131	0.154	0.515^∗∗^	0.001
LA area	0.333^∗∗^	0.001	0.615^∗∗^	0.001	0.116	0.207	0.386^∗∗^	0.001
LA length	0.172	0.060	0.413^∗∗^	0.001	0.172	0.061	0.292^∗∗^	0.001
LA diameter	0.246^∗∗^	0.007	0.465^∗∗^	0.001	0.111	0.228	0.230^∗^	0.012
*E*	-0.055	0.550	-0.009	0.925	0.011	0.907	0.076	0.411
*A*	0.181^∗^	0.049	0.178	0.053	-0.058	0.528	0.140	0.128
*E*/*A*	-0.183^∗^	0.046	-0.182^∗^	0.048	-0.031	0.738	-0.101	0.274

## Data Availability

The data used to support the findings of this study are available from the corresponding author upon request.

## Abbreviations

2c LAV: 2-chamber left atrial volume
2DE: Two-dimensional echocardiography
3D: Three-dimensional echocardiography
4c LAV: 4-chamber left atrial volume
AEF: Active emptying fraction
AEV: Active emptying volume
ASV: Active stroke volume
CCT: Cardiac computed tomography
CHD: Coronary heart disease
CHF: Congestive heart failure
CMR: Cardiac magnetic resonance
CT: Computed tomography
CVD: Cardiovascular disease
D1 group: Type 1 diabetic group
D2 group: Type 2 diabetic group
DBP: Diastolic blood pressure
DCM: Dilated cardiomyopathy
DD: Diastolic dysfunction
DM: Diabetes mellitus
*E*/*A*: Ratio of peak early to peak late mitral inflow velocity
*E*/*E*: Ratio of peak early mitral inflow to peak early diastolic tissue velocity of mitral annulus
EDV: End diastolic volume
HbA1C: Gylcated hemoglobin
HDL-C: High-density lipoprotein cholesterol
HF: Heart failure
LA: Left atrium
LAA: Left atrial appendage
LAAEV: Left atrial active emptying volume
LAEF: Left atrial ACTIVE emptying fraction
LAFV: Left atrial filling volume
LAPEV: Left atrial passive emptying volume
LASV: Left atrial total stroke volume
LAV: Left atrial volume
LAVi: Left atrial volume index
LDL: Low-density lipoprotein
LS: Longitudinal strain
LV: Left ventricle
LVEDd: Left ventricular end diastolic dimension
LVEF: Left ventricular ejection fraction
LVESd: Left ventricular end systolic dimension
LVM: Left ventricular mass
PALS: Peak atrial longitudinal strain
ROS: Reactive oxygen species
RT3DE: Real-time three-dimensional echocardiography
S: Seconds
SBP: Systolic blood pressure
SMI: Asymptomatic myocardial infarction
SR: Strain rate
SR-B: Scavengener receptor-B
SRI: Strain rate imaging
STE: Speckle-tracking echocardiography
TDI: Tissue Doppler imaging
TG: Triglycerides
TOE: Transesophageal echocardiography
TPA: Tissue plasminogen activator
TTE: Transthoracic echocardiography
*V*_max_: Left atrial maximum volume
*V*_min_: Left atrial minimum volume
*V*_preA_: Left atrial volume before atrial contraction.
